# IECata: interpretable bilinear attention network and evidential deep learning improve the catalytic efficiency prediction of enzymes

**DOI:** 10.1093/bib/bbaf283

**Published:** 2025-06-23

**Authors:** Jingjing Wang, Yanpeng Zhao, Zhijiang Yang, Ge Yao, Penggang Han, Jiajia Liu, Chang Chen, Peng Zan, Xiukun Wan, Xiaochen Bo, Hui Jiang

**Affiliations:** State Key Laboratory of NBC Protection for Civilian, No. 37, South Central Street, Changping District, Beijing 102205, China; School of Medicine, Shanghai University, No. 99, Shangda Road, Baoshan District, Shanghai 200444, China; Academy of Military Medical Sciences, No. 27, Taiping Road, Haidian District, Beijing 100039, China; State Key Laboratory of NBC Protection for Civilian, No. 37, South Central Street, Changping District, Beijing 102205, China; State Key Laboratory of NBC Protection for Civilian, No. 37, South Central Street, Changping District, Beijing 102205, China; State Key Laboratory of NBC Protection for Civilian, No. 37, South Central Street, Changping District, Beijing 102205, China; State Key Laboratory of NBC Protection for Civilian, No. 37, South Central Street, Changping District, Beijing 102205, China; State Key Laboratory of NBC Protection for Civilian, No. 37, South Central Street, Changping District, Beijing 102205, China; Shanghai Key Laboratory of Power Station Automation Technology, School of Mechatronics Engineering and Automation, Shanghai University, No. 99, Shangda Road, Baoshan District, Shanghai 200444, China; State Key Laboratory of NBC Protection for Civilian, No. 37, South Central Street, Changping District, Beijing 102205, China; Academy of Military Medical Sciences, No. 27, Taiping Road, Haidian District, Beijing 100039, China; State Key Laboratory of NBC Protection for Civilian, No. 37, South Central Street, Changping District, Beijing 102205, China

**Keywords:** *k*_cat_/*K*_m_ prediction, evidential deep learning, bilinear attention mechanism, uncertainty, interpretability

## Abstract

Enzyme catalytic efficiency (*k*_cat_/*K*_m_) is a key parameter for identifying high-activity enzymes. Recently, deep learning techniques have demonstrated the potential for fast and accurate *k*_cat_/*K*_m_ prediction. However, three challenges remain: (i) the limited size of the available *k*_cat_/*K*_m_ dataset hinders the development of deep learning models; (ii) the model predictions lack reliable confidence estimates; and (iii) models lack interpretable insights into enzyme-catalyzed reactions. To address these challenges, we proposed IECata, a *k*_cat_/*K*_m_ prediction model that provides uncertainty estimation and interpretability. IECata collected a dataset of 11 815 *k*_cat_/*K*_m_ entries from the BRENDA and SABIO-RK databases, along with an out-of-domain test dataset of 806 entries from the literature. By introducing evidential deep learning, IECata provides uncertainty estimates for *k*_cat_/*K*_m_ predictions. Moreover, it uses a bilinear attention mechanism to focus on learning crucial local interactions to interpret the key residues and substrate atoms in enzyme-catalyzed reactions. Testing results indicate that the prediction performance of IECata exceeds that of state-of-the-art benchmark models. More importantly, it provides a reliable confidence assessment for these predictions. Case studies further highlight that the incorporation of uncertainty in screening for highly active enzymes can effectively increase the hit ratio, thereby improving the efficiency of experimental validation and accelerating directed enzyme evolution. To facilitate researchers’ use of IECata, we have developed an online prediction platform: http://mathtc.nscc-tj.cn/cataai/.

## Introduction

Over the years, various techniques have been developed to enhance the enzyme-directed evolution process significantly. Methods such as error-prone PCR, DNA recombination, and site-directed saturation mutagenesis have shortened this process from millions of years in nature to just months in the laboratory [1–3]. However, the process of enzyme-directed evolution often requires multiple rounds of mutation and screening, and it remains a big challenge to obtain faster and more effective directed evolution strategies. The enzyme turnover number (*k*_cat_) and Michaelis constant (*K*_m_) are two key parameters for measuring the efficiency of enzyme-catalyzed reactions. Relying solely on *k*_cat_ or *K*_m_ makes it challenging to comprehensively evaluate the catalytic performance of an enzyme. However, *k*_cat_/*K*_m_ is a comprehensive evaluation parameter of enzyme catalytic efficiency, which can be used to compare the relative catalytic activities of different enzymes [4, 5]. Therefore, *k*_cat_/*K*_m_ can serve as a valuable guide for identifying high-activity enzymes, which could enhance the efficiency of the directed evolutionary screen for high-activity target enzymes.

Currently, the measurement of kinetic parameters of enzymes relies mainly on experiments, which are time-consuming, expensive, and labor-intensive, although the advancement of machine learning and deep learning has accelerated progress in fields like protein design and drug discovery [6–10]. Therefore, researchers have attempted to develop machine learning– and deep learning–based methods to accelerate the process of obtaining enzyme kinetic parameters [11–20]. For example, Heckmann *et al.* [11] used an integrated machine learning model to learn multiple input features such as metabolic network, protein structure, and substance concentration to achieve rapid prediction of *k*_cat_ in the *Escherichia coli* metabolic network, demonstrating the feasibility of the machine learning model to predict *k*_cat_. However, the input features of this model are too complex and difficult to apply for *k*_cat_ prediction in other species. Kroll *et al.* [12, 13] developed two organism-independent machine learning and deep learning models designed to rapidly predict the *K*_m_ and *k*_cat_ values of wild-type enzymes across different species. Li *et al.* [14] achieved high-throughput prediction of *k*_cat_ by using a recurrent neural network and graph neural network to learn information from amino acid sequences and a substrate molecular map, respectively. Kazuhiro Maeda *et al.* [16] proposed the MLAGO method, which achieved *K*_m_ prediction by employing five machine learning algorithms only based on three factors: the enzyme commission (EC) number, kyoto encyclopedia of genes and genomes (KEGG) compound ID, and organism ID. Yu *et al.* [15] developed a unified framework based on pretrained language models to predict *k*_cat_, *K*_m_, and *k*_cat_/*K*_m_. In addition, we developed a multitask learning–based model, MPEK, to predict *k*_cat_ and *K*_m_ simultaneously [21]. Although varieties of *k*_cat_ and/or *K*_m_ prediction tools have been developed, the values of *k*_cat_/*K*_m_ calculated based on the predicted *k*_cat_ and *K*_m_ often show deviations from experimental measurements. This deviation highlights the importance of developing a unified and reliable method for directly predicting *k*_cat_/*K*_m_.

However, there are three main challenges in predicting *k*_cat_/*K*_m_ using machine learning and deep learning methods. The first challenge is that there is no larger *k*_cat_/*K*_m_ dataset available. Existing studies, such as UniKP, only used 910 enzyme–substrate *k*_cat_/*K*_m_ entries. The insufficient amount of data limits the development of deep learning methods in the field of *k*_cat_/*K*_m_ prediction.

The second challenge is that existing models lack confidence estimations in their predictions. The limited availability of *k*_cat_/*K*_m_ data, particularly for certain enzyme types, makes it difficult for machine learning models to accurately capture the data distribution, leading to unreliable outputs and an underestimation of prediction errors when handling new, out-of-distribution (OOD) data. However, traditional deep learning models do not provide confidence estimates, resulting in a lack of reliable evaluation for their predictions.

The third challenge is how to focus on learning and explaining the crucial local interactions of enzyme-catalyzed substrates. The essence of enzyme catalysis is the interaction between crucial residues of the enzyme activity pocket and important substructures of the substrate molecule [22–24]. However, current models for predicting enzymatic reaction kinetic parameters often represent the reaction globally by concatenating or summing the feature vectors of the enzyme and substrate, without explicitly learning the crucial local interactions [11–15, 25–27]. This limitation not only limits the predictive performance of the model, but also hinders the interpretation of the predicted results.

The bilinear attention network is a very prospective technique for efficiently identifying local interactions between enzymes and substrates [28]. This method has been successfully applied in areas such as artificial intelligence–assisted drug design and visual question answering [29, 30]. On the other hand, uncertainty quantification (UQ) methods have shown excellent potential for assessing the confidence of prediction results. Existing UQ methods can be generally categorized into Bayesian approximation methods [31–35], ensemble methods [36–38], and similarly based methods [39, 40]. However, these methods require random sampling or similarity comparison to approximate the underlying uncertainty function. Although effective, these methods are more computationally expensive and take longer to run. Fortunately, Amini *et al.* and Sensoy *et al.* [41, 42] proposed an evidential deep learning (EDL) method that does not require sampling and can learn model prediction uncertainty without major modifications to the model architecture, enhancing both efficiency and flexibility. This method has been applied in fields such as molecular property prediction and image recognition, showing great promise for various applications [42–48].

Therefore, we propose a *k*_cat_/*K*_m_ prediction model with a bilinear attention network and EDL, named IECata. Firstly, IECata collected 11 815 *k*_cat_/*K*_m_ entries based on the BRENDA and SABIO-RK databases, which is ~13 times the size of the dataset used by UniKP. IECata used the ProtTrans (hereafter ProtT5) pretrained language model and light attention (LA) to extract enzyme features and a graph convolutional network (GCN) to extract substrate features. The bilinear attention network was used to learn the joint representation of enzyme and substrate, focusing on the local interactions between them. In addition, IECata used EDL for *k*_cat_/*K*_m_ prediction and estimated the uncertainty of the predicted values. The results demonstrate that learning the local key interactions between enzymes and substrates enhances the prediction performance of *k*_cat_/*K*_m_, surpassing the state-of-the-art (SOTA) model UniKP. This improvement is present in both the whole dataset using five-fold cross-validation (5CV) and the independent test dataset. The visualization of key residues and substrate atoms through the bilinear attention module provides interpretable insights into enzyme–substrate interactions and contributes to mechanistic studies of enzyme-catalyzed reactions. Through EDL, IECata assesses the reliability of predictions by providing a UQ for the result. More importantly, IECata can more accurately guide the screening of highly active target enzymes and accelerate the directed evolution of enzymes based on the *k*_cat_/*K*_m_ prediction and the uncertainty of the prediction.

**Figure 1:**
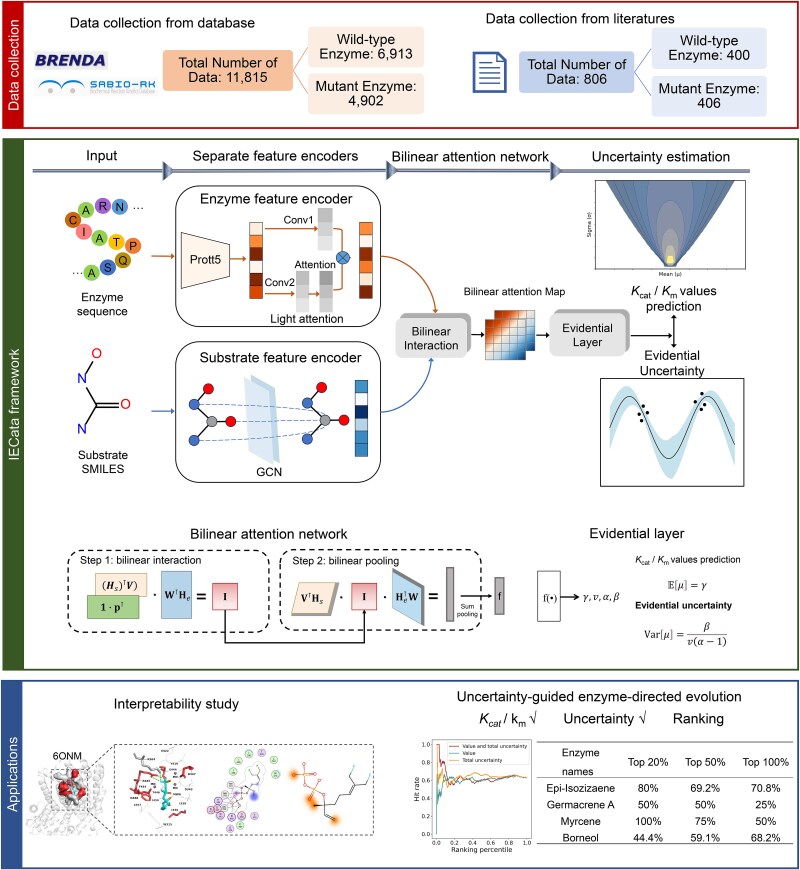
The framework of the proposed IECata.

## Results

### IECata framework

The proposed IECata framework is shown in Fig. 1. We first downloaded and processed the *k*_cat_/*K*_m_ dataset based on the BRENDA and SABIO-RK databases [49, 50]. A total of 11,815 *k*_cat_/*K*_m_ entries were collected, of which 6913 were wild-type enzymes and 4902 mutant enzymes. A total of 806 *k*_cat_/*K*_m_ entries were also collected from the literature, of which 400 were wild-type enzymes and 406 were mutant enzymes. Each entry contains the amino acid sequence of the enzyme, the simplified molecular input line entry system (SMILES) of the substrate, corresponding *k*_cat_/*K*_m_ values, and other information. Subsequently, representations of the enzyme and substrate were extracted. For the enzyme, the initial representation of the enzyme was extracted using the pretrained language model ProtT5 [51]. The initial representation of the enzyme was fed into the LA module [52] to further generate the task-relevant enzyme representation. The GCN was further used to encode the substrate topology information [53]. Next, the enzyme and substrate representations were fed into the bilinear attention network to generate a joint representation and to learn the local interactions between enzyme and substrate. Finally, the joint enzyme–substrate representation was fed into the EDL layer, outputting the predicted *k*_cat_/*K*_m_ values as well as the uncertainty of the prediction. The bilinear attention module visualizes the key residues and substrate atoms of enzyme-catalyzed substrates, providing interpretable insights into enzymatic reactions. When screening for high-activity enzymes, ranking enzymes by simultaneously considering predicted *k*_cat_/*K*_m_ values with uncertainty helps to reduce the false-positive rate of screening for high-activity enzymes.

### Data analysis

For IECata training and validation, we collected 11 815 entries based on *in vitro k*_cat_/*K*_m_ data stored in the BRENDA and SABIO-RK databases. To optimize model training, the raw labels were log10 transformed, resulting in a distribution that closely followed a normal pattern, primarily concentrated between −2 and 4 (Supplementary Fig. S1a). The dataset contains 6913 wild-type enzymes and 4902 mutant enzymes. Six class enzymes are included, with oxidoreductases being the majority and ligases being the least (Supplementary Fig. S1b). Compared with the UniKP dataset, which contains 910 *k*_cat_/*K*_m_ entries, IECata contains more entries and has a richer variety of enzymes and substrates (Supplementary Table S1).

We divided the dataset into a training dataset, validation dataset, and test dataset according to 8:1:1 and performed 10 repeated experiments. This independent test dataset is referred to as the in-domain independent test dataset, whose distribution is essentially the same as that of the training dataset (Supplementary Fig. S2a and b). In addition, an out-of-domain independent test dataset collected from the literature was used to test the performance of the model on the out-of-domain data. The out-of-domain independent test dataset does not appear in the training dataset, as shown in Supplementary Fig. S2d, the points of the out-of-domain test dataset are distributed outside the space of the training dataset. The distribution of the out-of-domain test dataset is significantly different from that of the training dataset (Supplementary Fig. S2a and c), with a *D* value and *P* value of 0.103 and 2.23 × 10^−4^ for the Kolmogorov–Smirnov test (K–S test) [54], respectively.

### High accuracy of IECata in in-domain test dataset

**Figure 2:**
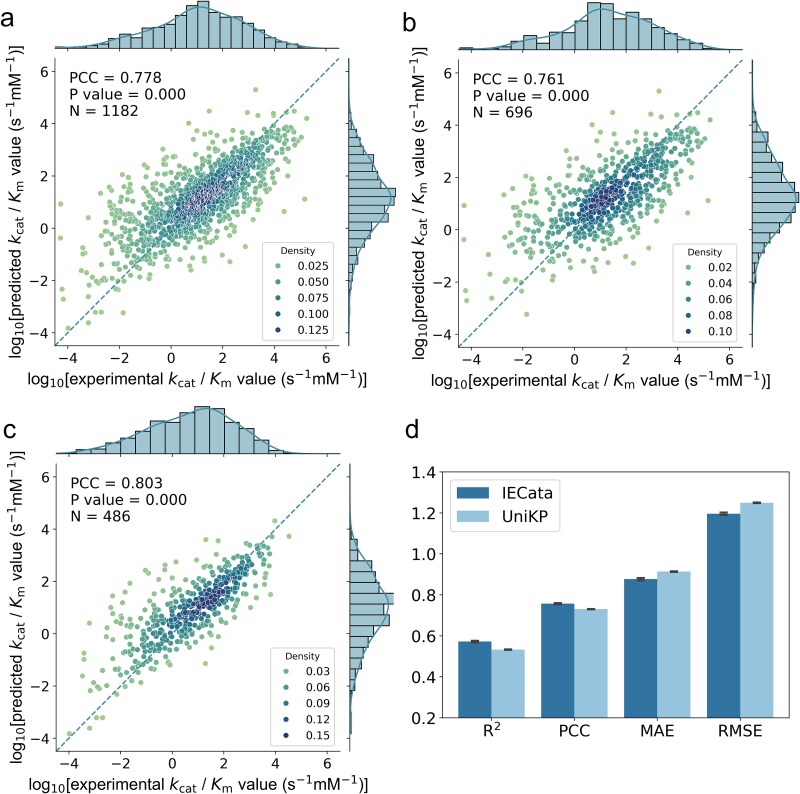
Performance of IECata on the in-domain test dataset. (a) Performance of IECata on the whole in-domain test dataset. (b) Performance of IECata on the in-domain test dataset of wild-type enzymes. (c) Performance of IECata on the in-domain test dataset of mutant enzymes. (d) Comparison of IECata’s and UniKP’s performances by 5CV on the whole dataset, *n* = 5 independent trials. The brightness of color represents the density of data points. Student’s *t*-test was used to calculate the *P* value for the PCC. *N* represents the number of entries in the test dataset.

We first explored the prediction performance for the IECata model on the in-domain test dataset. The models were trained with training and validation datasets and the saved model after training was tested on the in-domain test dataset. The Pearson correlation coefficient (PCC) for the in-domain test dataset reached 0.778 (Fig. 2a). According to the type of enzyme, the in-domain test dataset contained 696 wild-type enzyme entries and 486 mutant enzyme entries. IECata achieved a PCC of 0.761 for the wild-type enzymes and 0.803 for the mutant enzymes by independent testing (Fig. 2b and c).

To comprehensively evaluate the performance of IECata in predicting *k*_cat_/*K*_m_, we compared IECata with the current SOTA *k*_cat_/*K*_m_ prediction model, UniKP. Since UniKP used 5CV for model training and validation, IECata was also trained by 5CV on the whole dataset using the model hyperparameter trained on the 8:1:1 split dataset. The whole IECata dataset was fed into the UniKP model, tuned to the parameters, and then compared for performance (repeated 5 times with different random seeds). Figure 2d and Supplementary Table S3 compare the performance of IECata and UniKP. The average *R*-squared (*R*^2^), PCC, mean absolute error (MAE), and root mean square error (RMSE) of IECata reached 0.573, 0.757, 0.877, and 1.196, respectively, and the average *R*^2^, PCC, MAE, and RMSE of UniKP reached 0.533, 0.731, 0.914, and 1.250, respectively, indicating that IECata improved the *R*^2^ over UniKP by ~7.50% and the PCC by ~3.56% and reduced the MAE by ~4.05% and the RMSE by ~4.32%. To verify the statistical significance of these improvements, we performed *t*-tests. The results demonstrate statistically significant outperformance by IECata across the four valuation metrics (*P* < .05; Supplementary Table S3). These findings confirm that the performance enhancements achieved by IECata are not only substantial but also statistically significant.

In conclusion, IECata achieved good *k*_cat_/*K*_m_ prediction performance on the in-domain test dataset, as well as strong prediction performance on the wild-type and mutant enzymes. Compared with UniKP, IECata achieved superior prediction performance in several evaluation metrics.

### Ablation studies

To verify the effectiveness of the enzyme feature extraction via a pretrained model coupled with the LA mechanism, as well as the enzyme–substrate joint representation via bilinear attention, we carried out ablation studies comparing various enzyme feature extraction methods and enzyme–substrate joint representation methods.

#### Ablation studies on enzyme feature extraction methods

To explore the effectiveness of these encoded features in the proposed IECata, we considered three different methods for enzyme feature extraction: (i) Integer + convolutional neural networks (CNN), in which integer encoding was used to extract the enzyme sequence input features and a CNN was employed to extract the enzyme feature matrix [29]; (ii) ProtT5 + CNN, in which ProtT5 was employed to extract the enzyme input features and a CNN was further employed to transform the input features into task-relevant feature matrices; and (iii) ProtT5 + LA, in which ProtT5 was employed to extract the enzyme input features and LA was further employed to transform the input features into task-relevant feature matrices.

The above three enzyme feature encoding methods were used to test the prediction performance of IECata. Table 1 shows the average test results of the IECata model with 10 different random seeds. The average *R*^2^, PCC, MAE, and RMSE of ProtT5 + LA were 0.591, 0.771, 0.870, and 1.182, respectively. The average *R*^2^, PCC, MAE, and RMSE of ProtT5 + CNN were 0.536, 0.740, 0.912, and 1.259, respectively. The average *R*^2^, PCC, MAE, and RMSE of Integer + CNN were 0.506, 0.719, 0.951, and 1.299, respectively. ProtT5 + CNN performed better compared to Integer + CNN, indicating the effectiveness of ProtT5 in extracting the enzyme feature matrices. ProtT5 + LA achieved superior performance compared to ProtT5 + CNN, indicating that using LA to encode the enzyme initial feature matrix into a task-relevant feature matrix is more beneficial for extracting key information. Overall, the feature combination approach of ProtT5 + LA achieved superior *k*_cat_/*K*_m_ prediction performance and would be more suitable for *k*_cat_/*K*_m_ prediction.

**Table 1:** Test results of different enzyme feature extraction methods.

	*R* ^2^	PCC	MAE	RMSE
Integer + CNN	0.506 (0.020)^a^	0.719 (0.013)	0.951 (0.024)	1.299 (0.027)
ProtT5 + CNN	0.536 (0.018)	0.740 (0.012)	0.912 (0.025)	1.259 (0.024)
ProtT5 + LA^b^	**0.591** (0.010)	**0.771** (0.007)	**0.870** (0.013)	**1.182** (0.015)

^a^Standard deviation is indicated in parentheses.

^b^Bold indicates the optimal result for each evaluator.

#### Ablation studies on enzyme–substrate joint representation methods

To validate the effectiveness of bilinear attention, we performed ablation studies with different enzyme–substrate joint representations. We compared three different variants of enzyme–substrate joint representation: one-side enzyme attention, one-side substrate attention, and linear concatenation.

For one-side enzyme attention, the substrate representation and enzyme representation are updated by a learnable attention matrix, and the new enzyme representation is obtained by multiplying the attention coefficients with the enzyme representation [55]. One-side substrate attention is calculated similarly. The new substrate representation is obtained by multiplying the attention coefficients with the substrate representation. Linear concatenation is a simple vector concatenation of enzyme and substrate vector representations after a maximum pooling layer.

As shown by the results in Table 2, the bilinear attention method performs the best on all four metrics, having the highest *R*^2^ and PCC, and the lowest MAE and RMSE. This means that this method provides the most accurate prediction of *k*_cat_/*K*_m_ and the strongest correlation between predicted and true values. The performances of the one-side enzyme and one-side substrate attention methods are very close. They outperform the linear concatenation method, but are slightly lower than the bilinear attention method. In summary, the bilinear attention method is the optimal choice among these four methods, suggesting that using bilinear attention to focus on learning the crucial local interactions of the enzyme–substrate could help to improve the prediction performance of *k*_cat_/*K*_m_.

**Table 2:** Test results of different enzyme–substrate joint representation methods.

	*R* ^2^	PCC	MAE	RMSE
One-side enzyme attention	0.552 (0.011)^a^	0.755 (0.008)	0.884 (0.012)	1.236 (0.015)
One-side substrate attention	0.562 (0.008)	0.761 (0.004)	0.880 (0.010)	1.223 (0.011)
Linear concatenation	0.454 (0.086)	0.687 (0.061)	1.021 (0.084)	1.362 (0.104)
Bilinear attention^b^	**0.592** (0.011)	**0.771** (0.007)	**0.870** (0.013)	**1.180** (0.016)

^a^Standard deviation is indicated in parentheses.

^b^Bold indicates the optimal result for each evaluator.

### Evidential deep learning provides a reliable measure of uncertainty

Besides improving the prediction accuracy, providing reliable estimates of model prediction confidence is critical for guiding experimental decisions.

**Figure 3:**
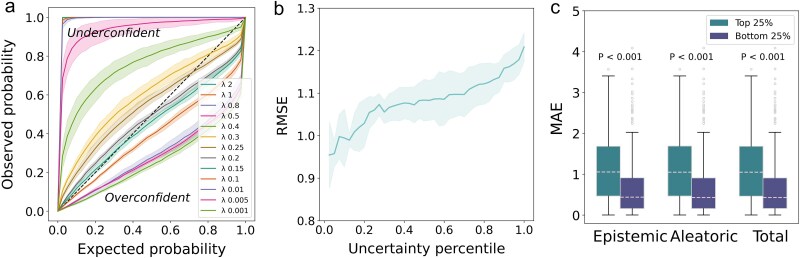
Uncertainty analysis of IECata on the in-domain test dataset. (a) Expected cumulative distribution against the observed cumulative distribution for IECata on the in-domain test dataset. The dashed line represents perfect calibration. Mean ± 95% CI, *n* = 5 independent trials. (b) RMSE at different uncertainty percentile cutoffs for IECata evaluated on the in-domain test dataset. Mean ± 95% CI, *n* = 5 independent trials. (c) Independent samples *t*-test on absolute error distributions for epistemic, aleatoric, and total uncertainty in two groups: top 25% uncertainty values and bottom 25% uncertainty values. All *P* values <.001.

First, we calibrated the evidential uncertainty on the in-domain test dataset. In the evidential regression model, the total loss function consists of two components: ${\mathcal{L}}^{\text{NLL}}(x)$ and ${\mathcal{L}}^R(x)$. The former represents the regression prediction loss. The latter measures the uncertainty loss, regulated by the regularization coefficient *λ*, which controls the strength of the uncertainty calibration [42, 43, 56, 57]. A perfect uncertainty estimate would lead to an exact match between the observed distribution and the ideally calibrated distribution. Using a perfectly calibrated classifier, we would expect to find the true target value within the 90% confidence interval 90% of the time. However, if the value of *λ* is smaller, the regression model will be overconfident and we will find the true value within the 90% confidence interval <90% of the time. If the value of *λ* is larger, the regression model will be underconfident and we will find the true value within the 90% confidence interval >90% of the time [44, 45, 48]. As shown in Fig. 3a, when *λ* = 0.2, a well-calibrated measure of uncertainty is provided. Thus, we chose *λ* = 0.2 for subsequent analyses.

In addition, we validated that the model can provide a reliable calibration of uncertainty by evaluating the relationship between prediction error and uncertainty. Fig. 3b shows that total uncertainty has a good positive correlation with the prediction error of the in-domain test dataset; i.e. samples with low uncertainty are more likely to achieve accurate prediction values. The total uncertainty comprises epistemic uncertainty and aleatoric uncertainty. Aleatoric uncertainty arises from inherent noise and variability in the data, while epistemic uncertainty stems from the limited knowledge of model parameters and predictions [58, 59]. To evaluate the model’s calibration to the epistemic uncertainty, aleatoric uncertainty, and total uncertainty, we performed independent-samples *t*-tests for absolute errors in the high- (top 25% uncertainty value) and low- (bottom 25% uncertainty value) uncertainty groups for the three uncertainties. Fig. 3c shows significant differences between the high- and low-uncertainty groups for the three uncertainties. The absolute error increases with increasing uncertainty, further confirming the strong correlation between evidential uncertainty and prediction error, thus demonstrating that IECata can provide a reliable calibration for prediction.

To quantitatively evaluate uncertainty calibration, two metrics—negative log-likelihood (NLL) and Spearman’s rank correlation coefficient—were employed (detailed in the online supplementary material). A comparison was made between the IECata-predicted uncertainty and that predicted by the model with random uncertainty (the IECata-predicted uncertainties were shuffled and randomly redistributed across the test set). This comparison helped to determine whether the uncertainty predicted by IECata is more reliable than random uncertainty.

**Figure 4:**
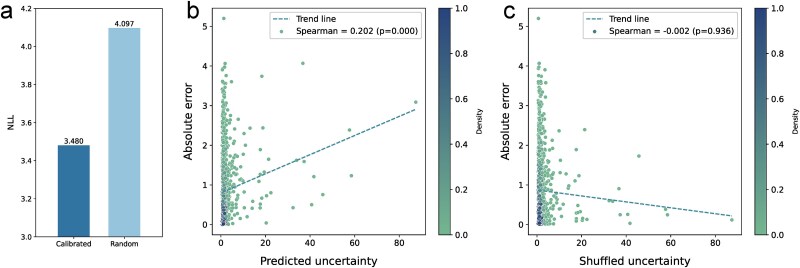
Quantitative assessment of uncertainty calibration. (a) Comparison of IECata and model with random uncertainty on NLL values. (b) The Spearman’s rank correlation coefficient value of IECata. (c) The Spearman’s rank correlation coefficient value of the model with random uncertainty.

NLL is employed to evaluate the correspondence between the predicted uncertainty distribution and the ground truth. IECata achieved an NLL of 3.480, significantly lower than the 4.097 obtained from the model with random uncertainty (Fig. 4a). Spearman’s rank correlation coefficient is employed to evaluate the monotonic relationship between prediction errors and uncertainty values. The uncertainty–error correlation yielded a coefficient of 0.202 (*P* < .001), indicating a statistically significant relationship (Fig. 4b). In contrast, the model with random uncertainty showed no significant correlation (coefficient = −0.002, *P* = .936; Fig. 4c). These results provide quantitative evidence demonstrating IECata’s effectiveness in uncertainty calibration.

### Uncertainty-enhanced IECata performance in out-of-domain test dataset

In the practical application of models, predictions for samples to be tested that have low similarity to the training dataset are usually not accurate enough. It is necessary to evaluate the effectiveness of models on out-of-domain datasets. To evaluate the prediction ability of IECata on out-of-domain data, we collected an independent test dataset based on the published literature, which contains 806 *k*_cat_/*K*_m_ entries after removing repetitions with the training dataset (see Methods for details of the collection process). The data distribution of this independent test dataset is significantly different from that of the training dataset, and the *t*-distribunted stochastic neighbor embedding (*t*-SNE) feature space is distributed outside the training dataset (Supplementary Fig. S2), referred to as the out-of-domain independent test dataset.

**Figure 5:**
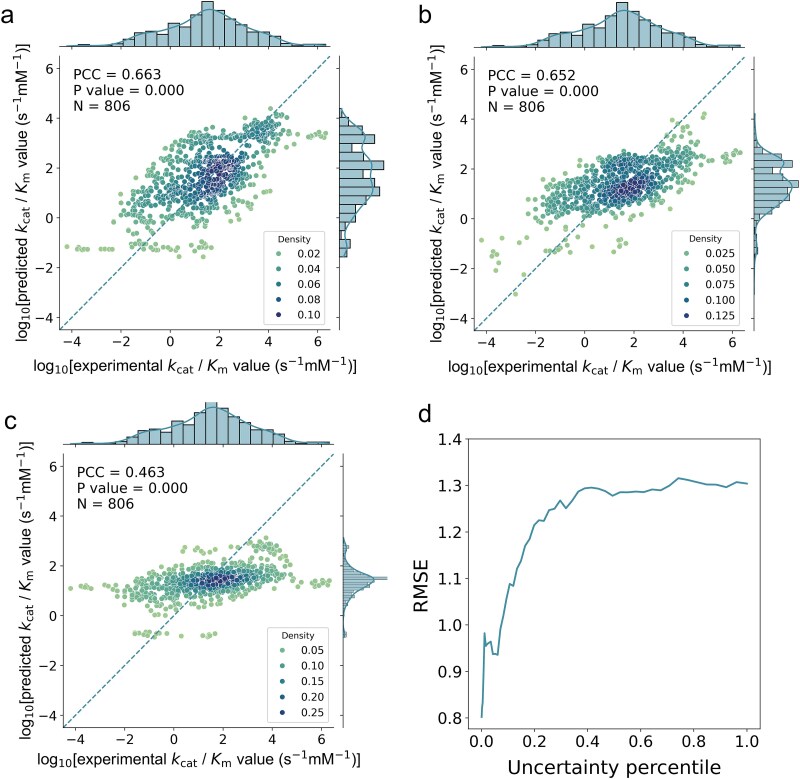
Performance of IECata and UniKP on the out-of-domain independent test dataset. (a) Performance of the IECata model on the out-of-domain independent test dataset. (b) Performance of the UniKP model, trained on the IECata training dataset, on the out-of-domain independent test dataset. (c) Performance of the model provided by UniKP on the out-of-domain test dataset. (d) RMSE at different uncertainty percentile cutoffs for IECata evaluated on the out-of-domain test dataset. The brightness of color represents the density of data points. Student’s *t*-test was used to calculate the *P* value for PCC. *N* represents the number of entries in the test dataset.

IECata achieved a PCC of 0.663 on the out-of-domain independent test dataset (Fig. 5a). The IECata training dataset was used to retune the parameters to train the UniKP model, and the saved model achieved a PCC of 0.652 when tested on the independent test dataset (Fig. 5b). When the out-of-domain independent test dataset was fed directly into the trained model provided by UniKP, which was trained using 5CV based on 910 *k*_cat_/*K*_m_ entries, the PCC was 0.463 (Fig. 5c). When the same training dataset was used for training IECata and UniKP models, the PCC of IECata tested on the out-of-domain independent test dataset was only slightly improved, by ~1.69%, over UniKP. On testing the models developed from IECata and UniKP using the out-of-domain independent test dataset, the results revealed that IECata outperformed UniKP significantly, with a PCC increase of ~43.2%. This indicates that using a larger training dataset enhances the model’s prediction ability, making IECata a more powerful *k*_cat_/*K*_m_ prediction model. More importantly, IECata can provide confidence estimates for predictions through uncertainty values. In the process of screening highly active enzymes, the success rate can be improved by the predicted *k*_cat_/*K*_m_ values with high confidence.

On analyzing the histogram of the distribution of IECata-predicted values and true values, it was found that the predicted values of *k*_cat_/*K*_m_ were mainly concentrated in the middle region. The predictions were not good for larger (log10-transformed *k*_cat_/*K*_m_ values > 5) or smaller (log10-transformed *k*_cat_/*K*_m_ values < −2) *k*_cat_/*K*_m_ values. This is mainly because there were fewer of these two types of entries in the training dataset, resulting in insufficient information for the model to learn. Therefore, the amount and richness of *k*_cat_/*K*_m_ data in the database need to be further improved.

Moreover, there was good correlation between RMSE and uncertainty in the out-of-domain test dataset, with the RMSE increasing rapidly as uncertainty increased up to 40% (Fig. 5d). This shows that predictions with lower uncertainty will have higher accuracy in the out-of-domain independent test dataset. Therefore, the introduction of uncertainty-assisted screening of high-activity target enzymes in the process of enzyme-directed evolution will improve the success rate, thus accelerating the process of enzyme-directed evolution.

### Uncertainty-guided enzyme-directed evolution

**Figure 6:**
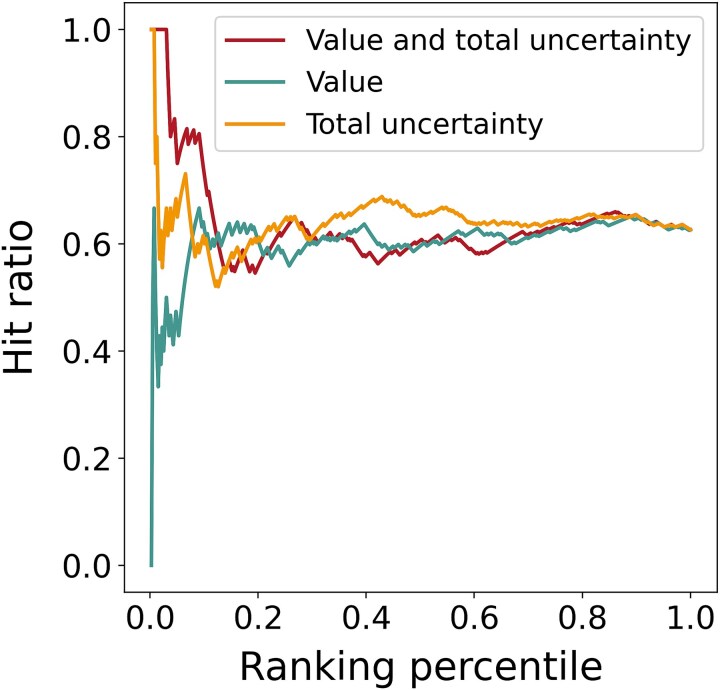
Comparing the predicted HRs of three different sorting strategies on the enzyme-directed evolution dataset.

To verify the guiding effect of IECata in enzyme-directed evolution, we further collated an enzyme-directed evolution dataset based on the out-of-domain independent test dataset. We labeled mutant enzymes “1” if their *k*_cat_/*K*_m_ value exceeded that of the corresponding wild-type enzyme, and “0” if it was lower. The directed evolution dataset contains 396 entries and was inputted into the IECata model. Three sorting strategies were employed to evaluate the hit ratio (HR) of the predicted labels: (i) sorting by predicted value plus uncertainty; (ii) sorting by uncertainty only; and (iii) sorting by predicted value only. The results in Fig. 6 show that sorting by predicted value plus uncertainty achieves a high HR in the top 20%. Beyond that point, sorting only by uncertainty achieved a higher HR. This highlights the significance of uncertainty in guiding enzyme-directed evolution screening. Additionally, both in-domain and out-of-domain test datasets revealed that predictions with lower uncertainty have higher accuracy. Therefore, we adopted the sorting method based on predicted value plus uncertainty, and mainly considered the top 20% of the predictions for subsequent enzyme-directed evolution screening.

We collected a directed evolution dataset for two sesquiterpene synthases and two monoterpene synthases from the published literature: (i) *epi*-isozizaene synthase (number of entries: *n* = 24) [60]; (ii) germacrene A synthase (*n* = 8) [61]; (iii) myrcene synthase (*n* = 8) [62]; and (iv) borneol synthase (*n* = 44) [63]. These data were not duplicated with the training dataset. The HRs of IECata predictions were calculated using a predicted value plus uncertainty ranking method. As shown in Table 3, *epi*-isozizaene synthase and germacrene A synthase both have higher HRs in the top 20%, outperforming the top 100%. Myrcene synthase achieved a 100% HR in the top 20%, better than the top 100%. However, borneol synthase only reached 44.4% in the top 20%. This indicates that while the IECata model effectively guides the directed evolution of sesquiterpene and monoterpene synthases by considering the prediction uncertainty, improving the accuracy for identifying high-activity target enzymes remains necessary.

### Interpretability of bilinear attention

#### Interpretability of bilinear attention visualization

**Figure 7:**
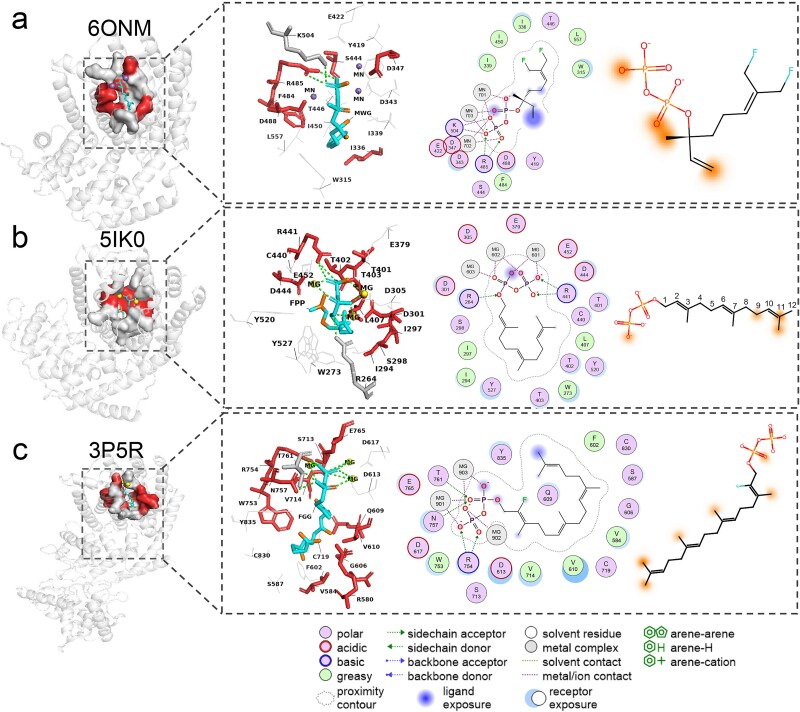
Visualization of substrates and binding pockets for interpretability study. The visualization results for 6ONM. From left to right, the surface structure of the 6ONM binding pocket, the predicted crucial residues within the pocket (labeled in blue for the substrate and in red for crucial residues), the 2D interaction maps of the binding pocket, and the crucial atoms of the substrate structure (labeled in orange for the crucial atoms). Binding pocket 3D structures were visualized using PyMOL. The 2D interaction maps were visualized using molecular operating environment (MOE) software, and all the substrate structures were visualized using RDKit. Panels (b) and (c) are the visualization results of structures 5IK0 and 3P5R, respectively.

IECata also excels at visualizing the contributions of each substructure to the predictions, making it easier to identify and interpret crucial residues and substrate atoms involved in enzyme–substrate interactions. This capability enhances our understanding of the mechanisms underlying these interactions. Here, we tested three cocrystallized structures of the enzyme and ligand: a monoterpene synthase [Protein Data Bank (PDB) ID: 6ONM], a sesquiterpene synthase (PDB ID: 5IK0), and a diterpene synthase (PDB ID: 3P5R) [64–66], each with a resolution > 3 Å. Moreover, the enzyme–substrate pairs were not present in the training dataset. The visualization results, displayed in Fig. 7, highlight the top 20% of weighted residues in red and the top 20% of weighted substrate atoms in orange within the 4.5 Å binding pocket. Additionally, enzyme–ligand 2D interaction diagrams from the corresponding X-ray structures are provided.

**Table 3:** Predicted HRs of IECata in the directed evolution datasets of sesquiterpene synthases and monoterpene synthases, based on the predicted *k*_cat_/*K*_m_ value plus uncertainty ranking.

Enzyme class	Enzyme name	Top 20% (%)	Top 50% (%)	Top 100% (%)
Sesquiterpene synthases	*epi*-Isozizaene	80	69.2	70.8
	Germacrene A	50	50	25
Monoterpene synthases	Myrcene	100	75	50
	Borneol	44.4	59.1	68.2

For the PDB structure 6ONM [64] [(+)-limonene synthase complexed with 8,9-difluorolinalyl diphosphate (DFGPP)], IECata correctly interpreted that the phosphate group is necessary for the DFGPP substrate to interact with limonene synthase (with residue R485 acting as a hydrogen bond donor and the oxygen-negative ion and oxygen of the phosphate group acting as a hydrogen bond acceptor). Similarly, the methyl group on the chiral and the carbon on the terminal alkene were also recognized as crucial atoms, which are clearly exposed to the solvent in the 2D interaction map (blue highlight), likely promoting further binding. Significantly, the conserved motif, DDXXD, is crucial for terpene synthases to generate terpenoids from substrates. IECata correctly identified D347 in this conserved motif as a crucial residue for the action of limonene synthase with DFGPP.

In the PDB structure 5IK0 [5-*epi*-aristolochene synthase with farnesyl pyrophosphate (FPP)] [65], interpretability again highlights the important interaction pattern of the substrate phosphate group with terpene synthase binding. Specifically, the oxygen-negative ion of the FPP substrate phosphate group formed a specific interaction with 5-*epi*-aristolochene synthase (with residue R441 acting as a hydrogen bond donor and the oxygen-negative ion of the phosphate group acting as a hydrogen bond acceptor). In contrast, the 9th methylene group, the 11th double-bond carbon, and the 11th methyl atom of FPP were incorrectly predicted to interact specifically with 5-*epi*-aristolochene enzyme, although slight exposure of the 11th methyl group to the solvent may promote further binding (blue highlights with weak extent). IECata failed to recognize the specific interaction of R264 with the substrate phosphate group, but correctly identified D301 in the conserved motif (DDXXD) as a crucial residue for the action of 5-*epi*-aristolochene synthase with FPP.

In the third case, the PDB structural complex (3P5R) of the taxadiene synthase complex with substrate 2-fluorogeranylgeranyl diphosphate (FGGDP) [66], the key role of the phosphate group of the substrate FGGDP was again highlighted and specific interactions formed by residues other than T761 were recognized (R754 and N757 acting as a hydrogen bond donor and the oxygen-negative ion and oxygen of the phosphate group acting as a hydrogen bond acceptor). However, the misrecognition of all the methyl groups of the substrate FGGDP is significant, although their partial exposure to the solvent may facilitate further binding.

Overall, the bilinear attention mechanism of the IECata model has a favorable explanatory potential for experimentally confirmed interactions. It is expected to reveal previously undiscovered local interactions, offering the possibility of unearthing hidden knowledge. These findings are expected to provide some guidance for enzyme-directed mutagenesis.

#### Quantitative validation of interpretability

Quantitative validation of the effectiveness of the bilinear attention mechanism for identifying key residues is crucial. To further validate the interpretability of IECata, two analyses were performed.

**Figure 8:**
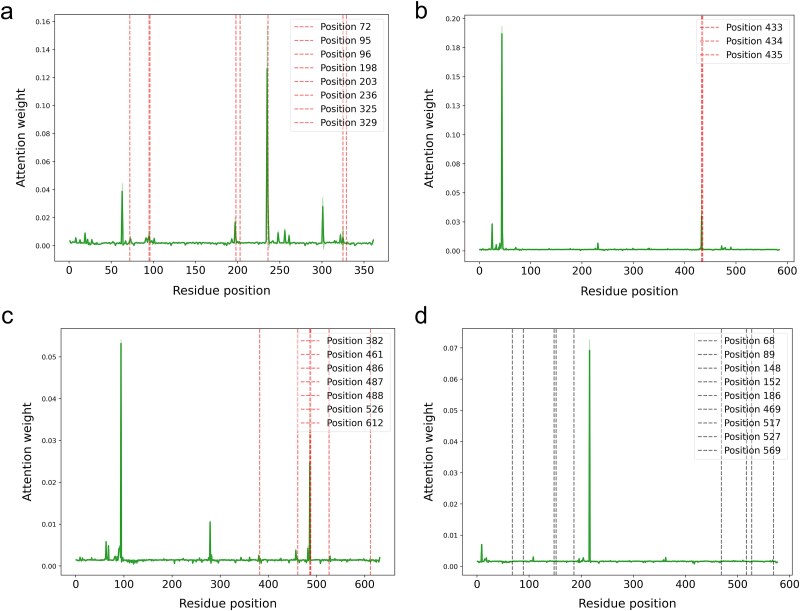
Validation of bilinear attention for identifying key residues in the enzyme-directed evolution datasets. (a) Attention weights for epi-isozizaene synthase mutant residues. (b) Attention weights for germacrene A synthase mutant residues. (c) Attention weights for borneol synthase mutant residues. (d) Attention weights for myrcene synthase mutant residues. Red dashed lines: key mutations with *k*_cat_/*K*_m_ or product yield changing by more than one magnitude from wild type. Black dashed lines: sub-key mutations with changes less than one magnitude from wild type. Solid curves: attention weights.

First, based on the directed evolution datasets of the four enzymes in the previous section, we analyzed the correspondence between residues with high attention weight and functional mutation sites that were experimentally validated. The highest attention weight residue of *epi*-isozizaene synthase overlapped with the key mutation site 236 (Fig. 8a). For germacrene A synthase, the second-highest attention weight residue coincided with the key mutation site 335 (Fig. 8b). Similarly, in borneol synthase, the second-highest attention weight residue aligned with position 448 (Fig. 8c). The myrcene synthase dataset contained only sub-key residue mutations that were predicted to have relatively small attentional weights (Fig. 8d). These results illustrate that IECata has a certain ability to recognize key residues.

**Figure 9:**
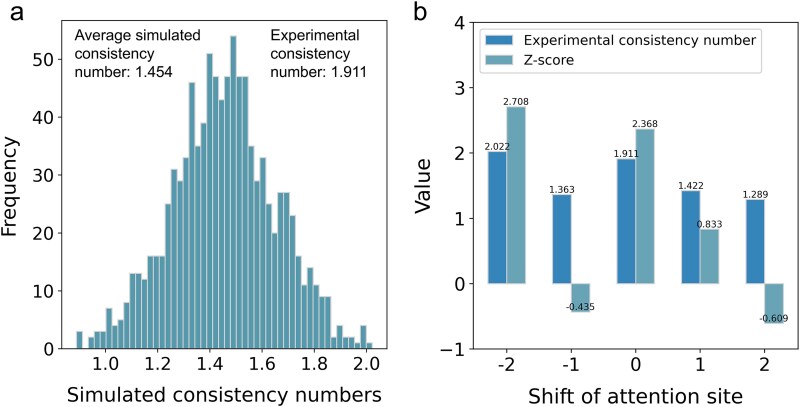
Quantitative validation of bilinear attention for identifying key residues. (a) Profile of simulated consistency numbers between the top 25 attention sites and randomly generated binding sites. (b) Effect of shifting attention sites on experimental consistency numbers and their *z*-score values.

Secondly, we compared the consistency of high-attention-weighted residues with experimentally determined binding sites. To achieve this, we constructed a dataset containing 45 sesquiterpene synthase structures based on the PDB database (see Methods for details). The amino acid sequences of each enzyme structure and the SMILES of the substrates were inputted to the IECata model to generate the corresponding attention weight matrices. Since the sesquiterpene synthase binding pocket in the dataset contains ~25 residues on average, we calculated the overlap between the top 25 high-attention residues and experimentally determined binding sites. This overlap count is referred to as the experimental consistency number. The mean of the experimental consistency number is 1.911 (Fig. 9a), indicating that on average about two predicted sites in each synthase structure correspond to the true binding site.

To evaluate the statistical significance of these findings, we conducted 1000 random sampling trials. In each trial, we randomly selected 25 residues to simulate a virtual binding pocket and calculated their overlap with high-attention residues (defined as the simulated consistency number). The statistical results showed that the average simulated consistency number was 1.454 (Fig. 9a), and the *z*-score (Supplementary Equation S7) calculated with the experimental consistency number (1.911) was 2.368, confirming that there was a certain correlation between the attention sites and the experimental binding sites.

To further validate the relationship between the high-attention residues and the experimental binding sites, we shifted the high-attention residues by −2, −1, 0, 1, and 2 to calculate the experimental consistency numbers and corresponding *z*-scores: 0 indicates the attention sites were not shifted, 1 indicates the attention sites were shifted one residue toward the C-terminal direction, and − 1 indicates the attention sites were shifted one residue toward the N-terminal direction. As shown in Fig. 9b, a shift of −2 increased the experimental consistency number and its corresponding *z*-score, whereas shifts of −1, 1, and 2 decreased these values. These results suggest that the attention mechanism is mainly focused on the exact binding site and the two residue neighboring sites at the C-terminus.

## Discussion

Directed evolution of enzymes to obtain high-activity enzymes is crucial for enhancing product yield and quality. Traditional methods for obtaining high-activity enzymes often require multiple rounds of mutation and screening, leading to long timelines, high costs, and low success rates. Currently, machine and deep learning methods can predict *k*_cat_/*K*_m_ values, significantly improving the efficiency of enzyme mutation and screening. However, existing methods face three main challenges: (i) the smaller size of the *k*_cat_/*K*_m_ dataset limits the application of deep learning in the field of *k*_cat_/*K*_m_ prediction; (ii) without UQ in *k*_cat_/*K*_m_ predictions, the model exhibits high prediction bias for high-value predictions, compromising the reliability of putative high-activity enzyme–substrate pairs; (iii) the key local enzyme–substrate interactions are not explicitly captured, limiting the performance and interpretability of the model. Therefore, this study first collected 11,815 *k*_cat_/*K*_m_ entries based on the BRENDA and SABIO-RK databases, and constructed a *k*_cat_/*K*_m_ prediction model utilizing EDL and bilinear attention based on the collected dataset, which predicts the *k*_cat_/*K*_m_ value while assessing the uncertainty (i.e. confidence) of the prediction. In the screening process for directed evolution of high-activity enzymes, enzyme–substrate pairs with higher predicted *k*_cat_/*K*_m_ values and lower uncertainty can be preferentially selected for experimental validation. Furthermore, IECata employs a bilinear attention mechanism to focus on crucial residues and crucial substrate atoms during the catalysis process. This enhances model performance and interprets key interactions during enzyme–substrate catalysis, providing valuable insights into the catalytic mechanisms of enzymes.

The results indicate that IECata outperforms the SOTA UniKP model in predicting *k*_cat_/*K*_m_. Significantly, IECata effectively calibrates prediction uncertainty, providing a robust measure of prediction confidence, and the bilinear attention mechanism has the capability of identifying key residues with substrate atoms. The innovative combination of EDL and the bilinear attention mechanism improves the accuracy, reliability and interpretability of *k*_cat_/*K*_m_ predictions, making IECata a powerful tool for screening highly active enzymes during enzyme-directed evolution.

However, a problem remains. As the available training data are still limited, the prediction accuracy needs to be improved for larger (log10-transformed *k*_cat_/*K*_m_ values > 5) or smaller (log10-transformed *k*_cat_/*K*_m_ values < −2) *k*_cat_/*K*_m_ values. Therefore, in future studies with limited *k*_cat_/*K*_m_ data, the loss-weighted and data-enhancement methods can be considered. Expanding the dataset by accumulating experimentally determined enzyme kinetic parameters and incorporating structural features of enzyme–substrate complexes would provide a more comprehensive representation of enzyme–substrate catalysis [67, 68]. Additionally, self-supervised learning techniques, such as contrastive learning and mutual information maximization, can effectively exploit the implicit information in unlabeled or multimodal data to learn deep connections between molecular structure and function without extensive manual annotation [69–71].

Finally, while EDL can calibrate the prediction uncertainty of unknown samples, it does not inherently improve prediction performance for OOD samples (i.e. cross-domain generalization). Meta-learning can address this limitation to some extent. Meta-learning models can acquire “prior knowledge” from previous learning experiences, allowing for rapid adaptation to new enzymes even with only a small amount of data [72–75]. This means that with the expansion of *k*_cat_/*K*_m_ data, even if there are only a few known catalytic efficiency data for a particular class of enzymes, potentially high-activity enzymes with similar catalytic efficiencies can be quickly identified. Combining EDL with techniques such as meta-learning or transfer learning can significantly enhance the efficiency of enzyme-directed evolution.

## Methods

### Data collection and processing

#### The whole dataset

The whole dataset was retrieved from the BRENDA and SABIO-RK enzymatic databases using a custom script on 1 March 2023 [49, 50]. Each entry included the EC number, substrate name, UniProt ID, enzyme type (wild type or mutant), organism, *k*_cat_/*K*_m_ value, and unit. To preserve the size and diversity of the training dataset, we excluded assay details like pH and temperature, as most *k*_cat_/*K*_m_ values from BRENDA and SABIO-RK lacked this information. We retrieved enzyme sequences from the UniProt database using the UniProt ID [76]. If an entry lacked a UniProt ID, we used the EC number and organism information to retrieve the enzyme’s sequence from the UniProt database. This approach helps to unambiguously identify the amino acid sequence of the bacteria. From the wild-type enzyme sequence, we derived the mutant enzyme sequence based on the corresponding mutation site. To avoid data redundancy caused by substrate homonyms, we used standard SMILES from PubChem to clean duplicate entries [77], which is essential to help filter redundant entries obtained from different databases. When entries are duplicated, the entry with the maximumt *k*_cat_/*K*_m_ value will be retained. In addition, entries were excluded for having strange units (e.g. ms^−1^, gs/L, and g/sL), enzyme sequences over 1200 amino acids, SMILES strings over 290 characters, or *k*_cat_/*K*_m_ values of 0. All *k*_cat_/*K*_m_ values were log10-transformed. The final dataset for constructing the IECata model comprised 11 815 high-quality entries, each including the EC number, substrate SMILES, enzyme sequence, enzyme type (wild type or mutant), log10-transformed *k*_cat_/*K*_m_ values, and unit (mM^−1^ s^−1^). Detailed data processing procedures and entries can be seen in Supplementary Fig. S3.

#### Out-of-domain independent test dataset

The out-of-domain independent test dataset was derived from literature published up to 20 August 2024 by searching the PubMed database for the keywords “*k*_cat_/*K*_m_” within the years 2000–24. This search retrieved 1300 articles, yielding 952 *k*_cat_/*K*_m_ entries through the manual collection. After removing duplicates within the entries and excluding overlaps with IECata’s training dataset and UniKP’s dataset, the final out-of-domain test dataset included 806 *k*_cat_/*K*_m_ entries. Each entry contained the EC number, organism, enzyme type (wild type or mutant), enzyme information (enzyme name or mutation information), UniProt ID, substrate, substrate SMILES, *k*_cat_/*K*_m_ value, unit (mM^−1^ s^−1^), PDB ID, and reference (PMID).

#### PDB structure dataset

To quantitatively validate the interpretability of IECata’s bilinear attention mechanism, we systematically curated a dataset of sesquiterpene synthase structures from the PDB. We initially identified 165 relevant structures using the “FPP” (the substrate of sesquiterpene synthase) as a search query on 10 April 2025. These structures are addressed according to the following criteria: (i) only structures with resolution ≤ 3.5 Å were retained to ensure structural reliability; (ii) only structures containing bound FPP substrates were retained to maintain biological relevance; and (iii) enzymes whose sequences were not present in the training dataset were retained to ensure an independent evaluation and avoid potential biases. The residues were located within 5 Å of the FPP substrate as the binding sites. Finally, we obtained 45 unique sesquiterpene synthase structures. These structures collectively contained 1114 binding sites, with each individual binding pocket comprising ~25 residues on average. Using this dataset, we performed a quantitative evaluation of IECata’s interpretability [8]. Specifically, we quantified the correspondence between the residues identified by IECata’s attention mechanism and the experimentally determined binding pocket residues through experimental consistency.

### Model construction

#### Molecular presentation

For enzymes, enzyme sequence information was extracted by the widely used protein language model ProtT5, a transformer-based self-supervised autocoder of protein sequences [51]. We extracted a 1024-dimensional sequence embedding for each residue, which resulted in a $1024\times L$ feature matrix for each enzyme, where $L$ represents the length of the enzyme sequence. We used ProtT5’s fixed parameters to derive these representations without further training.

Using the $1024\times L$-dimensional enzyme feature matrix as input $\bf x$, the task-relevant enzyme feature matrix was encoded via the LA [52]. First, two independent one-dimensional convolutions transformed the input to generate the attention coefficients and values *e*, *v*.


(1) \begin{equation*} {e}_{i,j}={b}_i+\sum\limits_{k=1}^{d_{\text{in}}}\sum\limits_{l=-\frac{s}{2}}^{\left\lceil \frac{s}{2}\right\rceil }{\bf W}_{\text{l,k,i}}^{({\text{e}})}{\boldsymbol{x}}_{k,j+l} \end{equation*}

where $L\times{d}_{\text{in}}$ is the input for LA, $b$ is the learned bias, $s$ is the filter size, and ${\bf W}^{(\text{e})}$ is the weight of the one-dimensional convolution.

To obtain the attentional weights, softmax normalization is required for${e}_{ij}$. The attention weight ${\alpha}_{ij}$ for the $j$th residue and the $i$th feature dimension is calculated as:


(2) \begin{equation*} {\alpha}_{i,j}=\frac{\exp \left({e}_{i,j}\right)}{\sum_{l=1}^l\exp \left({e}_{i,l}\right)} \end{equation*}

The weight distribution of each feature dimension $i$is independent and different weight distributions can produce different attention patterns. Subsequently, the attention vector for each residue is computed.


(3) \begin{equation*} {\boldsymbol{x}}_{i,j}={\alpha}_{i,j}{\boldsymbol{v}}_{i,j} \end{equation*}

The attention vector ${\boldsymbol{x}}_{i,j}$ was concatenated with the value vector ${\boldsymbol{v}}_{i,j}$ to obtain a feature vector for each residue with a dimension of 2048. To perform a bilinear attention operation with the substrate features, the feature vectors were dimensionality-reduced to 128 dimensions by a layer of multilayer perceptron. For the enzyme, we obtained a $128\times L$-dimensional feature matrix.

For the substrate, we constructed the substrate as a molecular graph and used the atomic information and topological information as the initial features of the node, including atom type, node degree, number of H atoms, and whether it is a chiral molecule or not. The specific feature-encoding process is described in the online supplementary material.

#### Bilinear attention network

Enzyme and substrate pairwise local interactions are captured using a bilinear attention network [29]. The bilinear attention network is a special form of cross-attention designed to explicitly model pairwise interactions between enzymes and substrates. It comprises two components: a bilinear interaction map to determine attention weights between pairs and a bilinear pooling layer on the interaction map to produce a joint enzyme–substrate representation.

Hidden representations for the enzyme and substrate, denoted as ${\mathbf{H}}_{\text{e}}=\left\{{\boldsymbol{h}}_{\text{e}}^1,{\boldsymbol{h}}_{\text{e}}^2,\dots, {\boldsymbol{h}}_{\text{e}}^L\right\}$ and ${\mathbf{H}}_d=\left\{{\boldsymbol{h}}_{\text{s}}^1,{\boldsymbol{h}}_{\text{s}}^2,\dots{\boldsymbol{h}}_{\text{s}}^N\right\}$, were generated using LA and GCN layers, where $L$represents the number of residues in the enzyme and $N$ represents the number of atoms in the substrate. First, two bilinear weight matrices $\mathbf{V}\mathbf{\in }{\mathbb{R}}^{D_{\text{s}}\times K}$ and $\mathbf{W}\mathbf{\in }{\mathbb{R}}^{D_{\text{e}}\times K}$ were used for substrate and enzyme representation learning. The learned hidden representations were then used to construct a bilinear interaction map, forming a single-headed pairwise interaction matrix **I**  $\in{\mathbb{R}}^{N\times L}$.


(4) \begin{equation*} \mathbf{Z}=\Big(\left(\mathbf{1}\bullet{\boldsymbol{p}}^{\intercal}\right)\circ{\sigma} \left(\left({\mathbf{H}}_{\text{s}}\Big){}^{\intercal}\mathbf{V}\right)\right) \end{equation*}

(5) \begin{equation*} \mathbf{I}=\mathbf{Z}\cdotp{\sigma} \left({\mathbf{W}}^{\intercal }{\mathbf{H}}_{\text{e}}\right) \end{equation*}

where $\boldsymbol{p}\mathbf{\in }{\mathbb{R}}^K$ is a learnable projection vector, $\mathbf{1}\mathbf{\in }{\mathbb{R}}^N$, and $\circ$ denotes the Hadamard product. Here, each element in $\mathbf{I}$ represents the interaction strength between the specific substructure pair of the enzyme and substrate.

To generate a joint representation ${\mathbf{f}}^{\prime}\in{\mathbb{R}}^K$, a bilinear pooling layer was applied to the interaction map $\mathbf{I}$. The $k$th element of${\mathbf{f}}^{\prime }$ was computed as follows:


(6) \begin{align*} {\mathbf{f}}_k^{\prime }=\text{ReLU}\left({\left({\mathbf{H}}_{\text{s}}\Big){}^{\intercal}\mathbf{V}\right)}_k^{\intercal}\bullet \mathbf{I}\bullet \text{ReLU}\right({\left({\mathbf{H}}_{\text{e}}\Big){}^{\intercal}\mathbf{W}\right)}_k\notag\\=\sum_{i=1}^N\sum_{j=1}^M{\mathbf{I}}_{\boldsymbol{i},\boldsymbol{j}}{\left({\boldsymbol{h}}_{\text{s}}^i\right)}^{\intercal}\left({\mathbf{V}}_k{\mathbf{W}}_k^{\intercal}\right){\boldsymbol{h}}_{\text{e}}^j \end{align*}

where ${\mathbf{V}}_k$ and ${\mathbf{W}}_k$ represent the $k$th column of the $\mathbf{V}$ and $\mathbf{W}$ matrices, respectively.

In addition, we applied sum pooling to the joint representation vector to obtain compact feature mappings:


(7) \begin{equation*} \mathbf{f}=\text{Sumpool}\left({\mathbf{f}}^{\prime },t\right) \end{equation*}

where $\text{Sumpool}\left(\bullet \right)$ is a one-dimensional nonoverlapping sum pooling operation with stride$t$, reducing ${\mathbf{f}}^{\prime}\in{\mathbb{R}}^K$ to $\mathbf{f}\mathbf{\in }{\mathbb{R}}^{K/t}$.

By computing multiple bilinear interaction mappings, we can extend a single bilinear interaction to a multihead form. The final joint representation vector is the sum of the individual heads. Since the weight matrices **V** and $\mathbf{W}$ are shared, each additional head only adds a new weight vector $\boldsymbol{P}$.

Therefore, through bilinear attention, the model explicitly learned enzyme–substrate pairwise interactions. To predict *k*_cat_/*K*_m_, we further inputted the joint representation $\mathbf{f}$into the evidential layer.

#### Evidential deep learning

For the *k*_cat_/*K*_m_ prediction task, we are given a dataset of paired training examples, where the labels are assumed to follow an independently identically distributed Gaussian distribution, $\theta =\left\{\mu, {\sigma}^2\right\}$, with unknown mean and variance. Here, the mean is assumed to follow a Gaussian distribution, while the variance follows an inverse gamma distribution. This structure yields a joint higher-order evidential distribution that conforms to the normal-inverse-gamma distribution [44, 78], specifically the normal-inverse-gamma distribution, $p\left(\theta |m\right)$, which is the conjugate prior for the Gaussian distribution. This distribution is parameterized by $m=\left\{\gamma, v,\alpha, \beta \right\}$ and denoted as the distribution over $\theta =\left\{\mu, {\sigma}^2\right\}$. In this work, we modify the final layer of the neural network to output the hyperparameters of the normal-inverse-gamma distribution. Therefore, for the *k*_cat_/*K*_m_ prediction task, the EDL has four output parameters: $\gamma, \kern0.5em v,\kern0.5em \alpha, \text{and}\ \beta$. Given the normal-inverse-gamma distribution, the predictions and uncertainties are derived from the moments of the distribution.


(8) \begin{equation*} \mathbb{E}\left[\mu \right]=\gamma \end{equation*}

(9) \begin{equation*} \text{Var}\left[\mu \right]=\frac{\beta }{v\left(\alpha -1\right)} \end{equation*}

where $\mathbb{E}\left[\mu \right]$represents the predicted value, and $\text{Var}\left[\mu \right]$ represents the predicted uncertainty.

Evidential models were trained using a dual-objective loss function, $\mathcal{L}(x)$, which consists of two loss terms: one for maximizing model fit through negative log-likelihood and an evidential regularizer to manage false evidence.


(10) \begin{equation*} \mathcal{L}(x)={\mathcal{L}}^{\text{NLL}}(x)+\lambda{\mathcal{L}}^R(x) \end{equation*}

where ${\mathcal{L}}^{\text{NLL}}(x)$ represents the negative log-likelihood loss, which measures the model’s fit to the data. ${\mathcal{L}}^R(x)$ denotes the evidential regularizer, which modulates the strength of the model’s evidence through the regularization coefficient $\lambda$. The $\lambda$ controls the balance between well-fitting data and the uncertainty confidence level. Higher values of $\lambda$ emphasize evidential regularization, resulting in more conservative and uncertain predictions.

#### Model evaluation and optimization

To comprehensively evaluate the predictive performance of the IECata regression model, we used four metrics: PCC, *R*^2^, MAE, and RMSE [79].The definitions for these evaluation metrics and the IECata optimization process and the final hyperparameters can be found in the online supplementary material (Table S2).

**Key Points**

- An evidential deep learning method, IECata, was proposed to predict *k*_cat_/*K*_m_ values and provide a reliable confidence assessment for these predictions.
- A bilinear attention mechanism was used to enhance the interpretability of key residues and substrate atoms in enzyme-catalyzed reactions.
- The proposed framework outperformed the existing state-of-the-art methods on the same test datasets.
- An online server was provided to predict *k*_cat_/*K*_m_ values easily and efficiently.

## Supplementary Material

Supplementary_bbaf283

## Data Availability

The IECata web server is freely available at http://mathtc.nscc-tj.cn/cataai/. The model saved after training can be found at https://github.com/zhaoyanpeng208/IECata. All the relevant data supporting the results of this study are provided in the Source data. All the codes for IECata dataset collection and processing, model training, validation, and testing are available at https://github.com/zhaoyanpeng208/IECata.
