# Supplementary material for: IECata: interpretable bilinear attention network and evidential deep learning improve the catalytic efficiency prediction of enzymes
Source: Brief Bioinform. 2025 Jun 23;26(3):bbaf283. doi: 10.1093/bib/bbaf283 (PMC12205960; doi:10.1093/bib/bbaf283)
Supplement: Supplementary_bbaf283 [file supplementary_bbaf283.docx]

# IECata: Interpretable bilinear attention network and evidential deep learning improve the catalytic efficiency prediction of enzymes

Jingjing Wang ^1^ **^¶¶^**, Yanpeng Zhao^2, 3^ **^¶¶^**, Zhijiang Yang ^1^, Ge Yao ^1^, Penggang Han ^1^, Jiajia Liu ^1^, Chang Chen ^1^, Peng Zan^4^ *****, Xiukun Wan ^1^ *****, Xiaochen Bo^3^ *, Hui Jiang ^1^ *****

____________

The authors state no conflict of interest.

**^¶¶^** These authors contributed equally to this work.

^1^ State Key Laboratory of NBC Protection for Civilian, Changping District, Beijing, China

^2^ School of Medicine, Shanghai University, Baoshan District, Shanghai, China

^3^ Academy of Military Medical Sciences, Haidian District, Beijing, China

^4^ Shanghai Key Laboratory of Power Station Automation Technology, School of Mechatronics Engineering and Automation, Shanghai University, Baoshan District, Shanghai, China

* Corresponding authors

E-mails: zanpeng@shu.edu.cn (PZ), xiukunwan@126.com (XW), boxiaoc@163.com; boxc@bmi.ac.cn (XB), ylplkmc@163.com; jiangtide@sina.cn (HJ).

### Model optimization and evaluation

The model was trained using the Adam optimizer and a dual-objective loss function $\mathcal{L}\left( x \right)$to minimize the loss. Hyperparameter tuning focused on the regularization parameter $\lambda$, learning rate, batch size, epochs, and dropout rate to optimize RMSE and PCC on validation datasets. Finally, the regularization parameter $\lambda$ of 0.2, the batch size of 64, the learning rate of 8e^-4^, the epochs of 100, and the fixed step learning rate decay (StepLR) with a step size of 30 and a decay factor of 0.5. More parameters and the detailed tuning process are shown in Table S2, with the final settings in bold. IECata was trained on a NVIDIA HGX A100 with 80GB of memory.

To comprehensively evaluate the predictive performance of the IECata regression model, we used four metrics: Pearson coefficient (PCC), R-squared (R^2^), mean absolute error (MAE), and root mean square error (RMSE).

|  | $\mathrm{PCC}=\frac{\sum_{i=1}^{n} \left( y_{ie}-\bar{y}_{e} \right)\left( y_{ip}-\bar{y}_{p} \right)}{\sqrt{\sum_{i=1}^{n} \left( y_{ie}-\bar{y}_{e} \right)^{2}}\sqrt{\sum_{i=1}^{n} \left( y_{ip}-\bar{y}_{p} \right)^{2}}}$ | (1) |
| --- | --- | --- |
|  | $R^{2}=1-\frac{\sum_{i=1}^{n} {(y_{ie}-y_{ip})}^{2}}{\sum_{i=1}^{n} {(y_{ie}-\bar{y}_{e})}^{2}}$ | (2) |
|  | $\mathrm{MAE}=\frac{1}{n}\sum_{i=0}^{n} \left\vert y_{ip}-y_{ie} \right\vert$ | (3) |
|  | $\mathrm{RMSE}=\sqrt{\frac{1}{n}\sum_{i=1}^{n} {(y_{ip}-y_{ie})}^{2}}$ | (4) |

Where,$y_{ie} \mathrm{and} y_{ip}$are the experimental and predicted *k*_cat_ / *K*_m_ values, respectively. $\bar{y}_{e}$and $\bar{y}_{p}$are the average of the experimental and predicted *k*_cat_ / *K*_m_ values, respectively. $n$ is the number of entries in the datasets.

The hit ratio (HR) was used to measure the proportion of enzyme-directed evolution data correctly predicted by IECata.

|  | $HR=\frac{1}{N}\sum_{i=1}^{N} hit(i)$ | (5) |
| --- | --- | --- |

$N$ is the number of samples. $hit\left( i \right)$whether the predicted labels match the true labels in enzyme-directed evolution dataset. If yes, its value is 1, else it is 0.

The negative log likelihood (NLL) and the Spearman's rank correlation coefficient were employed to quantitatively evaluate uncertainty calibration. NLL evaluates how well the predicted uncertainty distribution matches the ground truth by measuring the "surprise" of observing the true data under the model’s probabilistic predictions:

|  | $\mathrm{NLL}=-\sum_{i=1}^{n} \log p(y_{ie}\vert\mathbb{E}\left[ \mu\right],\mathrm{Var}\left[ \mu\right])$ | (6) |
| --- | --- | --- |

where $y_{ie}$ is the true *k*_cat_ / *K*_m_ value and $\mathbb{E}\left[ \mu\right],\mathrm{Var}\left[ \mu\right]$ are the predicted *k*_cat_ / *K*_m_ value and uncertainty of the EDL prediction. The smaller the NLL value, the more closely the predictive distribution fits the real data.

Spearman's rank correlation coefficient is a non-parametric measure of the strength of the monotonic relationship between two variables. It calculates the degree of association between two variables by comparing their rank order (i.e., their rank in the dataset). Spearman's rank correlation coefficient has a value between -1 and 1. if the coefficient value is close to 1, it indicates a positive correlation. A coefficient value close to -1 indicates a negative correlation. When the coefficient is close to 0, it indicates that the relationship between the two variables is insignificant or there is no monotonic relationship.

The z-score is a statistical concept used to represent the difference between a specific value and the mean, measured in terms of standard deviations. Its standard definition is:

|  | $Z=\frac{X-\mu}{\sigma}$ | (7) |
| --- | --- | --- |

Where $X$ is the experimental consistency number; $\mu$ is the mean of the simulated consistency number; $\sigma$ is the standard deviation of the simulated consistency number.

The z-score standardizes data with different scales or units, making it easier to compare values. It indicates how many standard deviations a particular value is from the mean. A z-score of 0 means the value is exactly at the mean; a positive z-score indicates the value is above the mean, while a negative z-score indicates it is below the mean.

**Substrate molecule representation**

The classical GCN is used as the substrate encode. The GCN extracts information from the neighboring nodes of each node, and this process is achieved by calculating a weighted summation. The current node features are then summed with the aggregated features of the neighboring nodes and activated by a nonlinear activation function, and finally the node features are normalized using the BatchNorm function thus obtaining the node features of the next layer. Finally, each substrate molecule is represented as a $128\times N$-dimensional feature matrix, where $N$ is the number of atoms contained in the substrate.

|  | $h_{i}^{(l+1)}=\text{BN}(\sigma(b^{(l)}+h_{i}^{l}+\sum_{j\in N(i)} \frac{1}{c_{ij}}h_{j}^{(l)}W^{l}))$ | (8) |
| --- | --- | --- |
|  | $c_{ij}=\sqrt{\left\vert D(j) \right\vert}\sqrt{\left\vert D(i) \right\vert}$ | (9) |

Where $b^{(l)}$ and $W^{l}$ are the model parameters of GCN, which can be obtained by training and learning. $h_{i}^{l}$ is the feature vector of graph node $i$ in layer $l$. $N(i)$ is the dataset of neighbor nodes of graph node $i$. $j\in N(i)$ represents that graph node $j$ is a neighbor node of graph node $i$. $D(i)$ and $D(j)$ are the in-degree of graph nodes $i$ and $j$, respectively. $c_{\mathrm{ij}}$ is the product of the square roots of the in-degree of graph nodes $i$ and $j$. $\sigma$ is the non-linear activation function, the ReLU function. BN is the BatchNorm function, which normalizes layer inputs to avoid problems like vanishing and exploding gradients.

# Figures


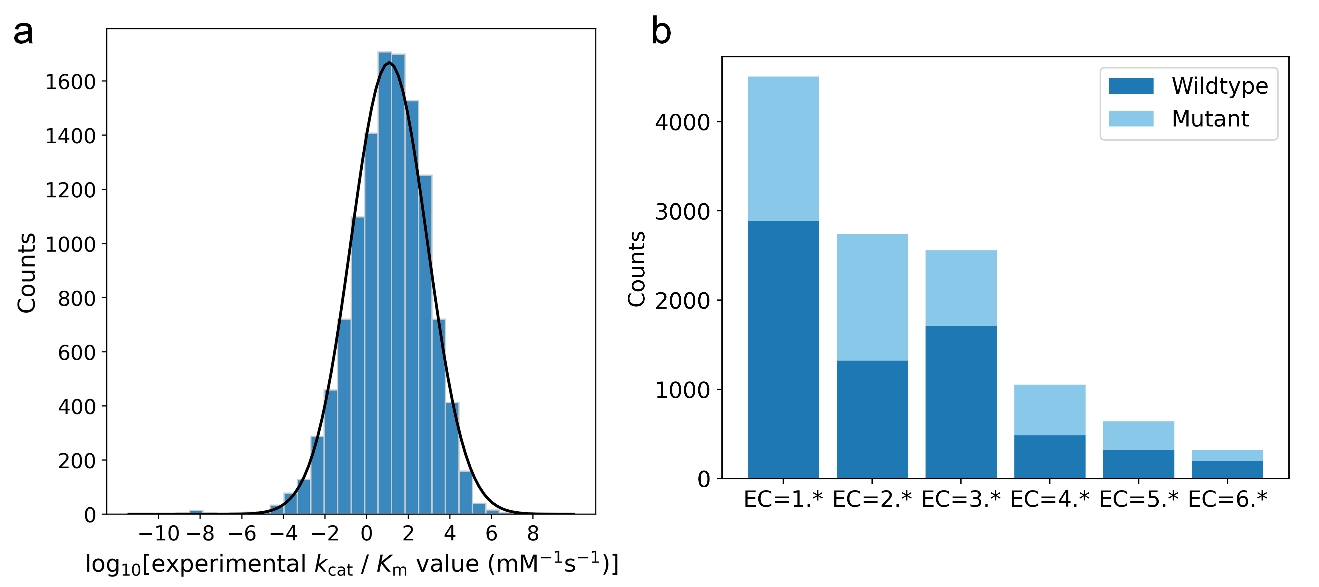


**Figure S1.** Data analysis of the whole IECata dataset. (a) Data distribution of all *k*_cat_ / *K*_m_ values. (b) Classification of enzyme types based on the first digit of EC number, and the distribution of wildtype and mutant enzymes for each type of enzyme.


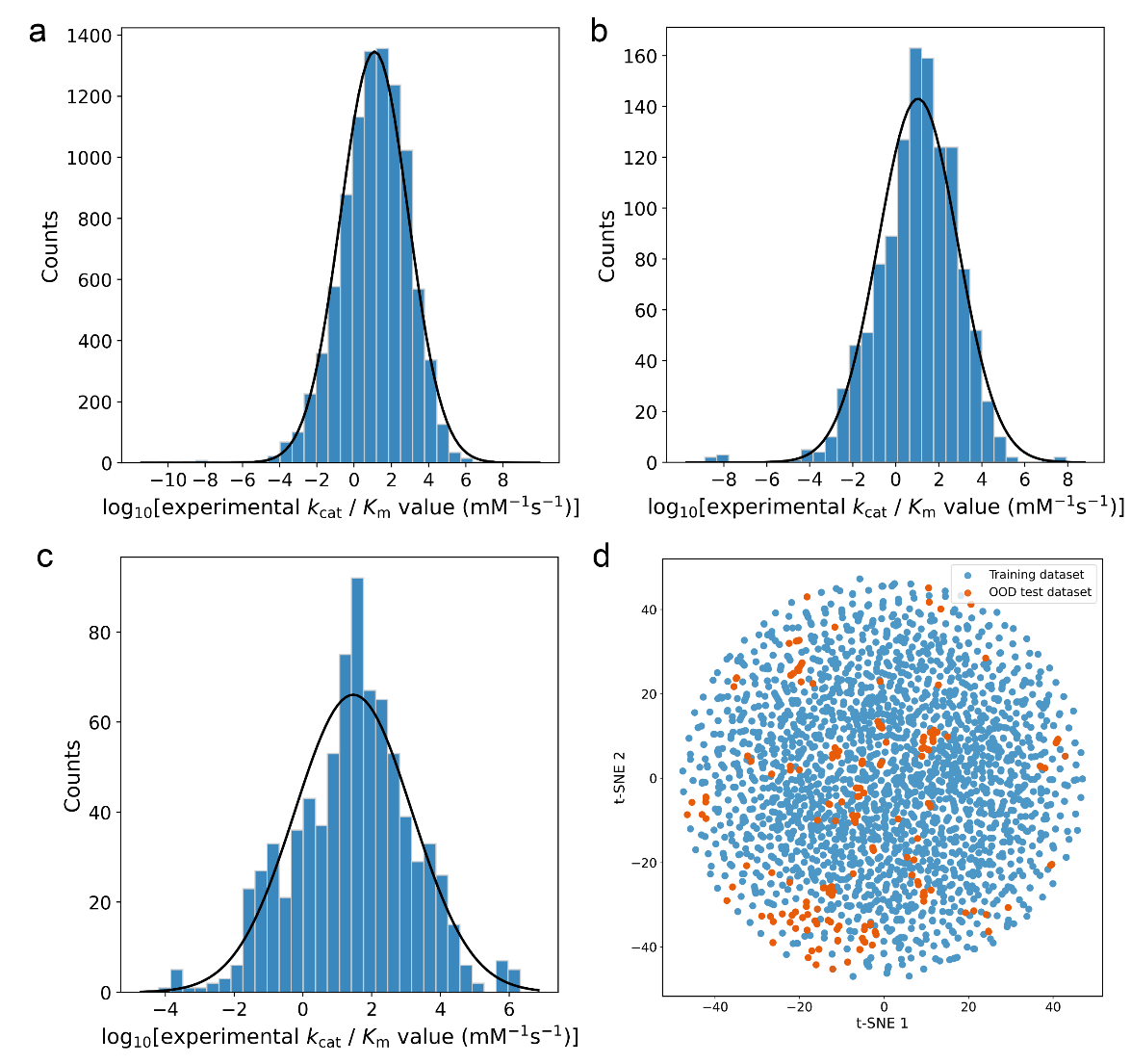


**Figure S2.** Distribution analysis of the IECata dataset. **a** The distribution of IECata train dataset. **b** The distribution of IECata test dataset split from the whole dataset (in-domain test dataset). **c** The distribution of IECata test dataset collected from the literature (out-of-domain test dataset). **d** t-SNE visualization of the sample space of the training dataset and OOD test dataset, revealing that the OOD test dataset was beyond the training distribution.


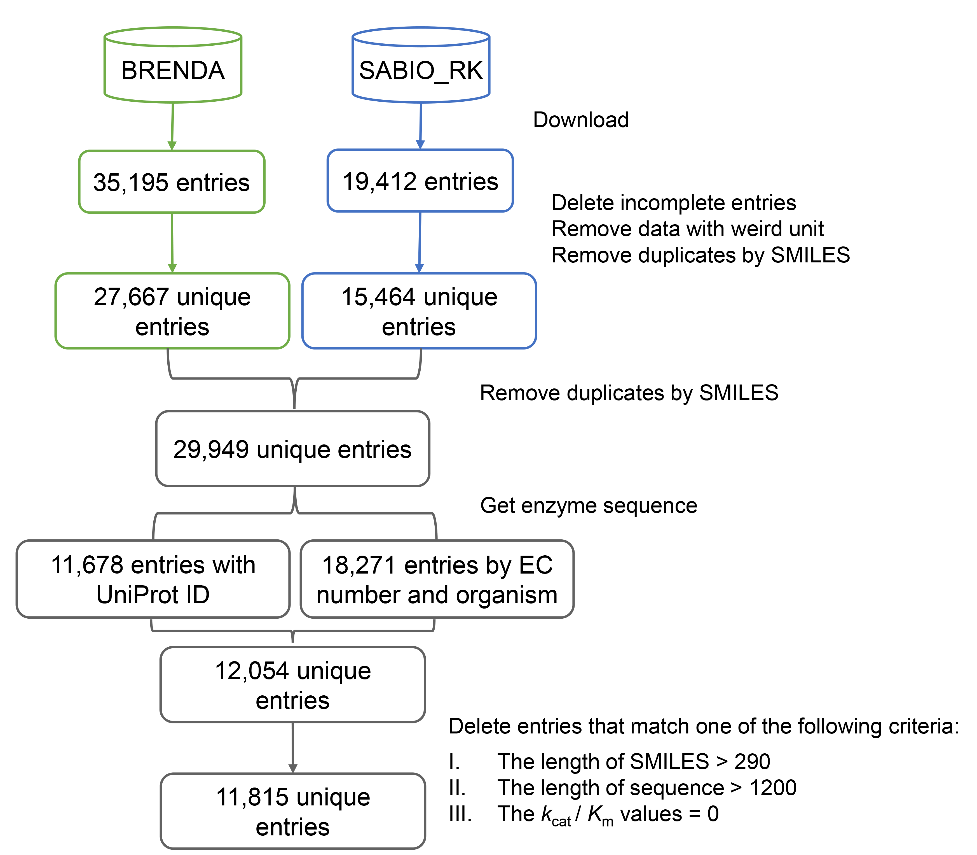


**Figure S3.** The collection and processing workflow of the IECata dataset.

# Tables

**Table S1.** Comparison of IECata and UniKP datasets.

| *k*_cat_ / *K*_m_ datasets | IECata | UniKP |
| --- | --- | --- |
| Entries | 11,815 | 910 |
| Unique enzyme sequences | 5,350 | 321 |
| Unique Enzyme Classes (EC) | 1,279 | NA |
| Unique substrates | 2,113 | 452 |
| Unique organisms | 901 | NA |

**Table S2.** Hyperparameter settings for the IECata model.

| Parameters | Values |
| --- | --- |
| Regularization coefficient $\lambda$ | 0.005, 0.01, 0.1, 0.15, **0.2**, 0.25, 0.3, 0.4, 0.5 |
| Epoch | 80, 85, 90, 95, **100**, 110, 120, 130, 140, 150 |
| Batch | 16, 32, **64**,128, 256 |
| Optimizer | **Adam** |
| Learning rate | 1e^-5^, 5e^-5^, 5e^-4^, 6e^-4^, 7e^-4^, **8e^-4^**, 1e^-3^, 5e^-3^ |
| Weight_decay by StepLR | **Step 30, Gamma 0.5**  Step 25, Gamma 0.5  Step 20, Gamma 0.5 |
| Dropout | 0.1, 0.15, **0.2**, 0.3, 0.35, 0.4 |
| Enzyme feature convolution kernel size | **9** |
| Enzyme attention convolution kernel size | **9** |
| Enzyme readout MLP layers | **1** |
| Enzyme readout MLP hidden dimension | 64, **128**, 256 |
| Substrate GCN layers | 2, **3**, 4 |
| Substrate GCN hidden dimension | [64, 64,64]  **[128,128,128]**  [256,256,256] |
| Enzyme substrate pair readout MLP layers | **4** |
| Enzyme substrate pair readout MLP hidden Dimension | **[256,512,128,4]**  [256,256,128,4]  [256,128,128,4]  [512,256,128,4] |

The final hyperparameters are indicated in bold.

**Table S3** Performance comparison of IECata and UniKP on the whole dataset by 5CV.

| **Metrics** | **UniKP** | **IECata** | **Improvement** | **p-value** |
| --- | --- | --- | --- | --- |
| R²↑ | 0.533±0.001 | 0.573±0.004 | 7.50% | 3.75×10⁻^8^ |
| PCC↑ | 0.731±0.001 | 0.757±0.003 | 3.56% | 3.53×10⁻^8^ |
| RMSE↓ | 1.250±0.002 | 1.196±0.006 | 4.05% | 7.05×10⁻^7^ |
| MAE↓ | 0.914±0.002 | 0.877±0.006 | 4.32% | 4.07×10⁻^8^ |
